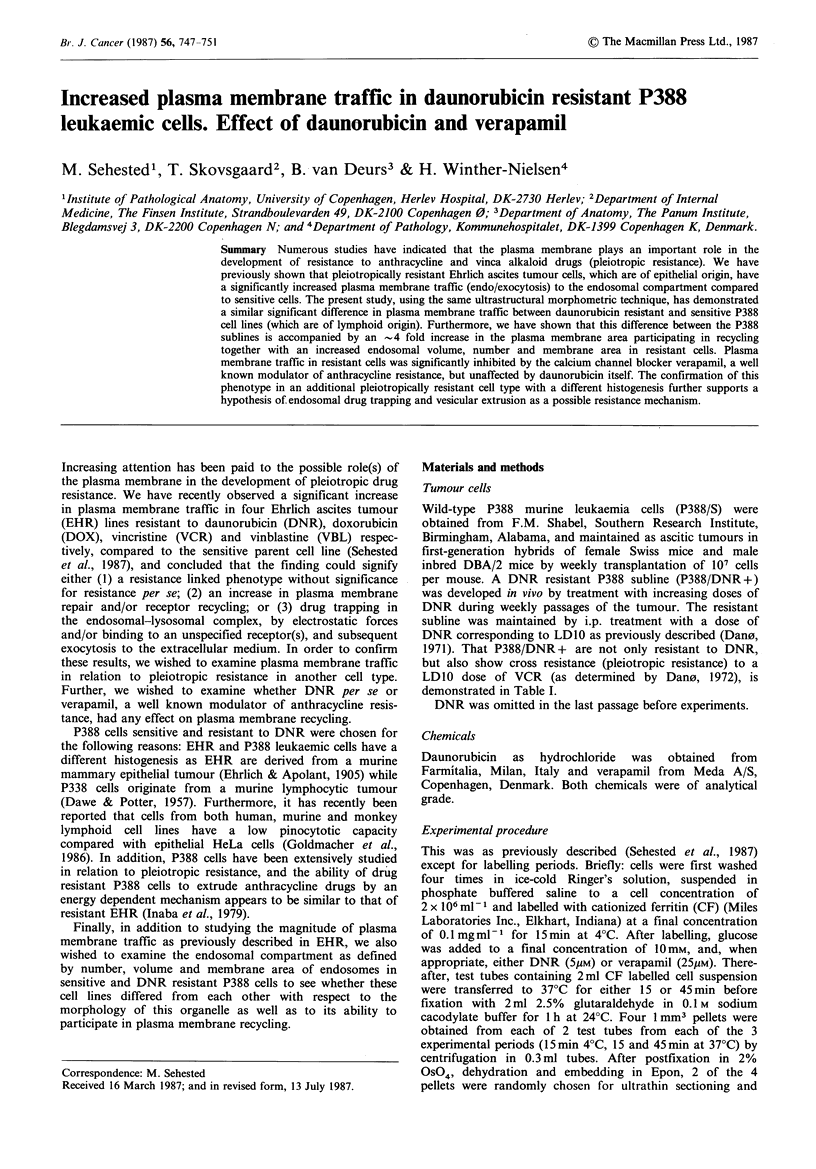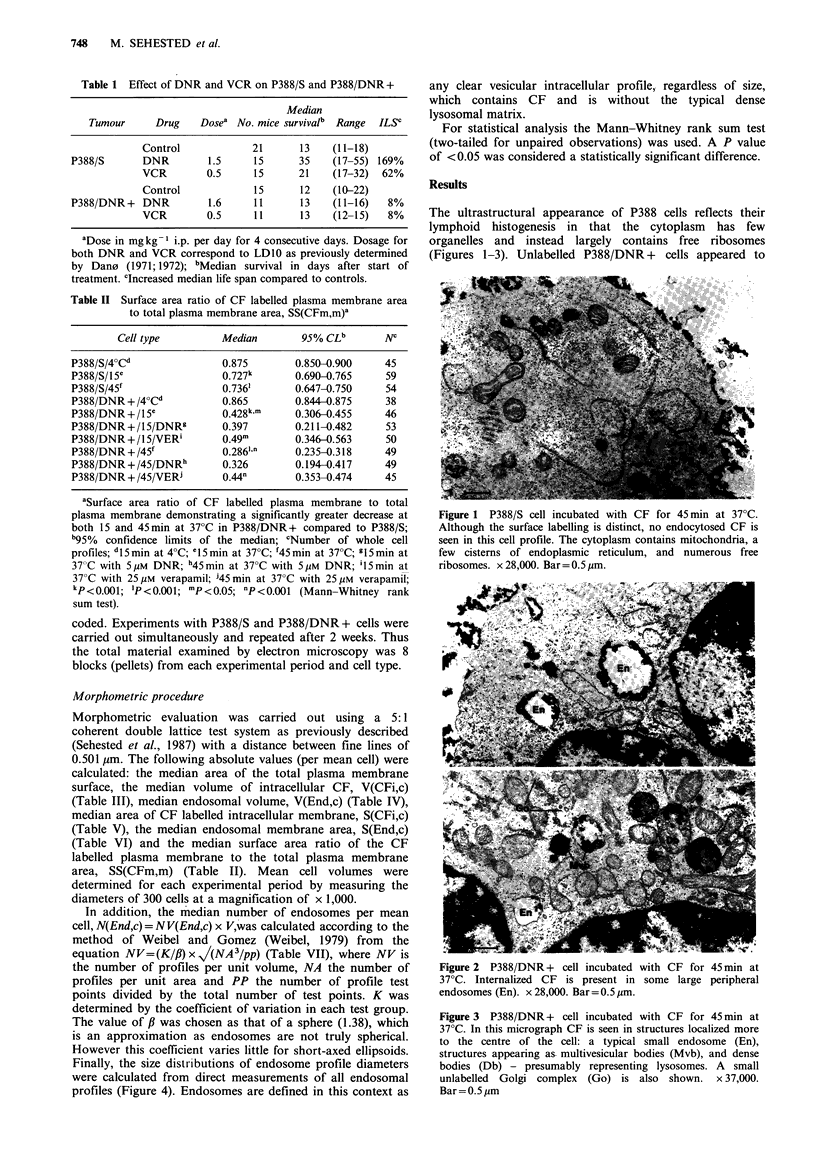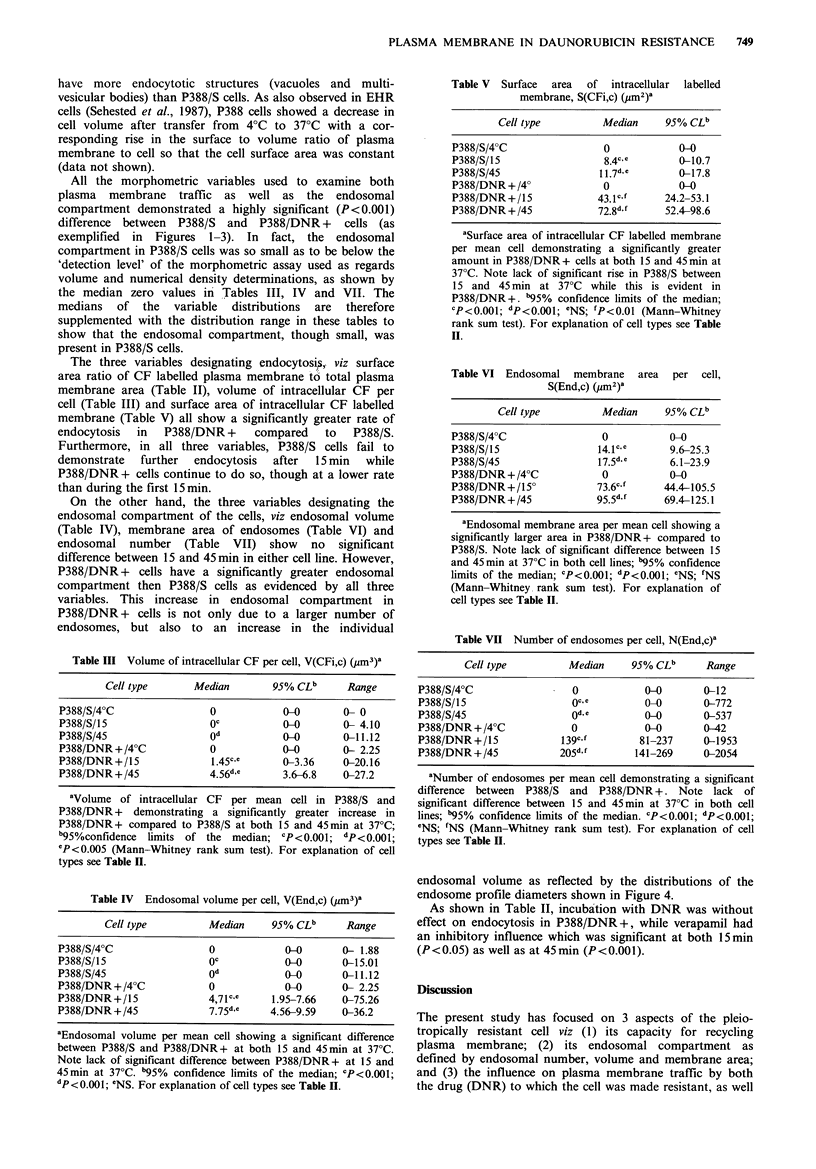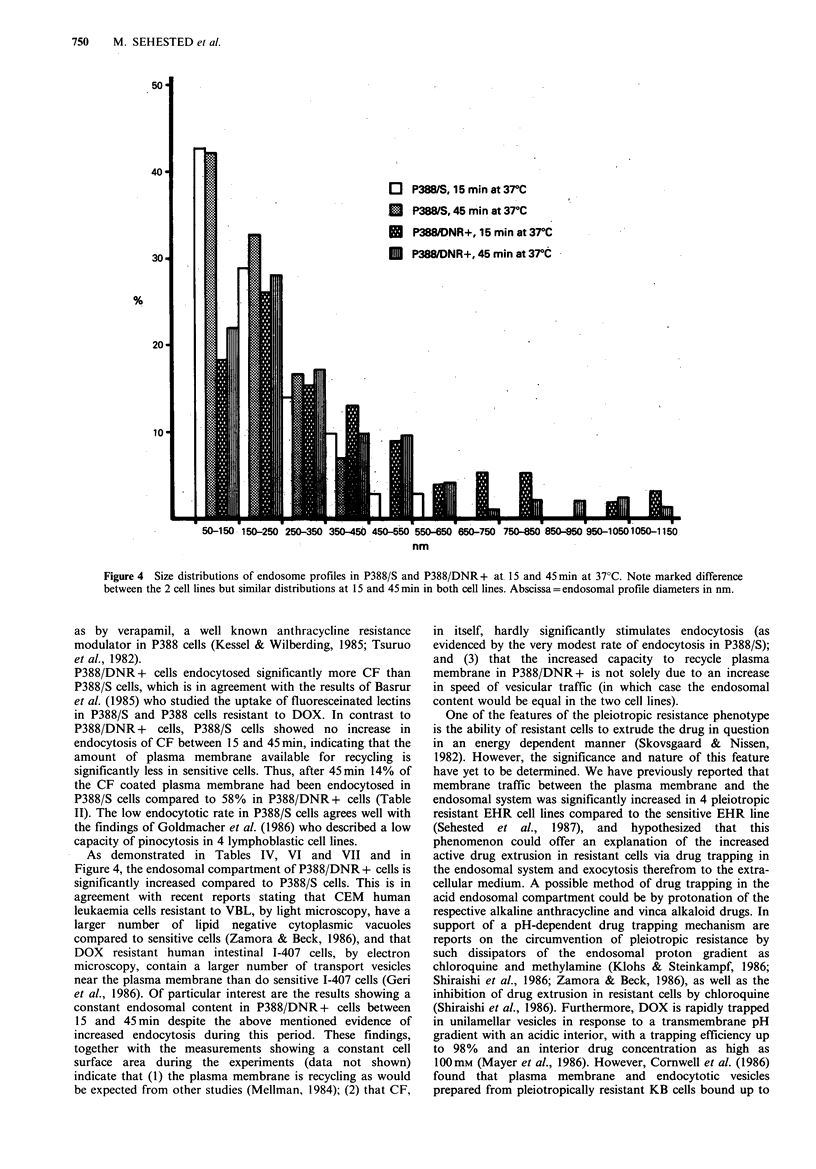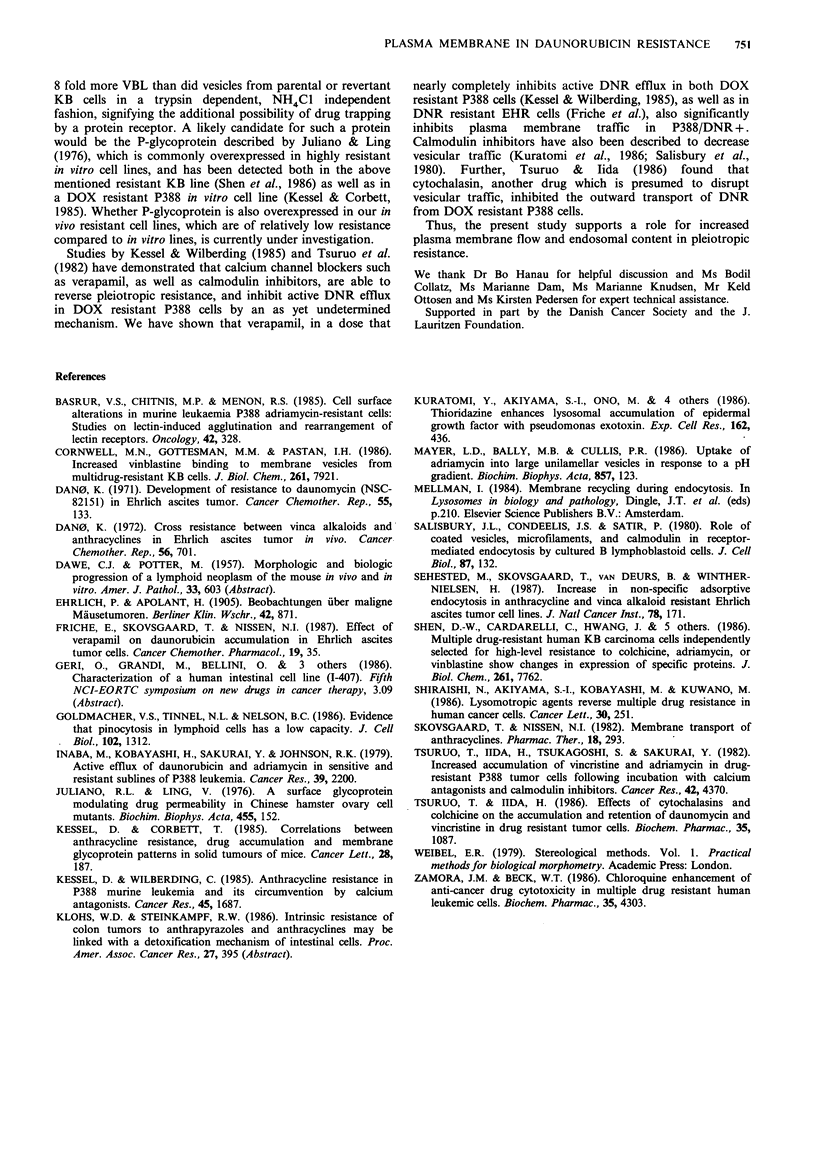# Increased plasma membrane traffic in daunorubicin resistant P388 leukaemic cells. Effect of daunorubicin and verapamil.

**DOI:** 10.1038/bjc.1987.282

**Published:** 1987-12

**Authors:** M. Sehested, T. Skovsgaard, B. van Deurs, H. Winther-Nielsen

**Affiliations:** Institute of Pathological Anatomy, University of Copenhagen, Herlev Hospital, Denmark.

## Abstract

**Images:**


Br. J. Cancer (1987) 56, 747–751 © The Macmillan Press Ltd., 1987

### Increased plasma membrane traffic in daunorubicin resistant P388 leukaemic cells. Effect of daunorubicin and verapamil

M. Sehested[1], T. Skovsgaard[2], B. van Deurs[3] & H. Winther-Nielsen[4]

[1]*Institute of Pathological Anatomy, University of Copenhagen, Herlev Hospital, DK-2730 Herlev;* [2]*Department of Internal Medicine, The Finsen Institute, Strandboulevarden 49, DK-2100 Copenhagen Ø;* [3]*Department of Anatomy, The Panum Institute, Blegdamsvej 3, DK-2200 Copenhagen N; and* [4]*Department of Pathology, Kommunehospitalet, DK-1399 Copenhagen K, Denmark.*

**Summary** Numerous studies have indicated that the plasma membrane plays an important role in the development of resistance to anthracycline and vinca alkaloid drugs (pleiotropic resistance). We have previously shown that pleiotropically resistant Ehrlich ascites tumour cells, which are of epithelial origin, have a significantly increased plasma membrane traffic (endo/exocytosis) to the endosomal compartment compared to sensitive cells. The present study, using the same ultrastructural morphometric technique, has demonstrated a similar significant difference in plasma membrane traffic between daunorubicin resistant and sensitive P388 cell lines (which are of lymphoid origin). Furthermore, we have shown that this difference between the P388 sublines is accompanied by an ~4 fold increase in the plasma membrane area participating in recycling together with an increased endosomal volume, number and membrane area in resistant cells. Plasma membrane traffic in resistant cells was significantly inhibited by the calcium channel blocker verapamil, a well known modulator of anthracycline resistance, but unaffected by daunorubicin itself. The confirmation of this phenotype in an additional pleiotropically resistant cell type with a different histogenesis further supports a hypothesis of endosomal drug trapping and vesicular extrusion as a possible resistance mechanism.

Increasing attention has been paid to the possible role(s) of the plasma membrane in the development of pleiotropic drug resistance. We have recently observed a significant increase in plasma membrane traffic in four Ehrlich ascites tumour (EHR) lines resistant to daunorubicin (DNR), doxorubicin (DOX), vincristine (VCR) and vinblastine (VBL) respectively, compared to the sensitive parent cell line (Sehested *et al.*, 1987), and concluded that the finding could signify either (1) a resistance linked phenotype without significance for resistance *per se*; (2) an increase in plasma membrane repair and/or receptor recycling; or (3) drug trapping in the endosomal–lysosomal complex, by electrostatic forces and/or binding to an unspecified receptor(s), and subsequent exocytosis to the extracellular medium. In order to confirm these results, we wished to examine plasma membrane traffic in relation to pleiotropic resistance in another cell type. Further, we wished to examine whether DNR *per se* or verapamil, a well known modulator of anthracycline resistance, had any effect on plasma membrane recycling.

P388 cells sensitive and resistant to DNR were chosen for the following reasons: EHR and P388 leukaemic cells have a different histogenesis as EHR are derived from a murine mammary epithelial tumour (Ehrlich & Apolant, 1905) while P338 cells originate from a murine lymphocytic tumour (Dawe & Potter, 1957). Furthermore, it has recently been reported that cells from both human, murine and monkey lymphoid cell lines have a low pinocytotic capacity compared with epithelial HeLa cells (Goldmacher *et al.*, 1986). In addition, P388 cells have been extensively studied in relation to pleiotropic resistance, and the ability of drug resistant P388 cells to extrude anthracycline drugs by an energy dependent mechanism appears to be similar to that of resistant EHR (Inaba *et al.*, 1979).

Finally, in addition to studying the magnitude of plasma membrane traffic as previously described in EHR, we also wished to examine the endosomal compartment as defined by number, volume and membrane area of endosomes in sensitive and DNR resistant P388 cells to see whether these cell lines differed from each other with respect to the morphology of this organelle as well as to its ability to participate in plasma membrane recycling.

Correspondence: M. Sehested
Received 16 March 1987; and in revised form, 13 July 1987.

### Materials and methods

### *Tumour cells*

Wild-type P388 murine leukaemia cells (P388/S) were obtained from F.M. Shabel, Southern Research Institute, Birmingham, Alabama, and maintained as ascitic tumours in first-generation hybrids of female Swiss mice and male inbred DBA/2 mice by weekly transplantation of $10^7$ cells per mouse. A DNR resistant P388 subline (P388/DNR+) was developed *in vivo* by treatment with increasing doses of DNR during weekly passages of the tumour. The resistant subline was maintained by i.p. treatment with a dose of DNR corresponding to LD10 as previously described (Danø, 1971). That P388/DNR+ are not only resistant to DNR, but also show cross resistance (pleiotropic resistance) to a LD10 dose of VCR (as determined by Danø, 1972), is demonstrated in Table I.

DNR was omitted in the last passage before experiments.

### *Chemicals*

Daunorubicin as hydrochloride was obtained from Farmítalia, Milan, Italy and verapamil from Meda A/S, Copenhagen, Denmark. Both chemicals were of analytical grade.

### *Experimental procedure*

This was as previously described (Sehested *et al.*, 1987) except for labelling periods. Briefly: cells were first washed four times in ice-cold Ringer's solution, suspended in phosphate buffered saline to a cell concentration of $2 \times 10^6 ml^{-1}$ and labelled with cationized ferritin (CF) (Miles Laboratories Inc., Elkhart, Indiana) at a final concentration of $0.1 mg ml^{-1}$ for 15 min at 4°C. After labelling, glucose was added to a final concentration of 10 mM, and, when appropriate, either DNR (5 μM) or verapamil (25 μM). Thereafter, test tubes containing 2 ml CF labelled cell suspension were transferred to 37°C for either 15 or 45 min before fixation with 2 ml 2.5% glutaraldehyde in 0.1 M sodium cacodylate buffer for 1 h at 24°C. Four $1 mm^3$ pellets were obtained from each of 2 test tubes from each of the 3 experimental periods (15 min 4°C, 15 and 45 min at 37°C) by centrifugation in 0.3 ml tubes. After postfixation in 2% $OsO_4$, dehydration and embedding in Epon, 2 of the 4 pellets were randomly chosen for ultrathin sectioning and

**Table I** Effect of DNR and VCR on P388/S and P388/DNR+

| *Tumour* | *Drug* | *Dose*[a] | *No. mice* | *Median survival*[b] | *Range* | *ILS*[c] |
|---|---|---|---|---|---|---|
| | Control | | 21 | 13 | (11–18) | |
| P388/S | DNR | 1.5 | 15 | 35 | (17–55) | 169% |
| | VCR | 0.5 | 15 | 21 | (17–32) | 62% |
| | Control | | 15 | 12 | (10–22) | |
| P388/DNR+ | DNR | 1.6 | 11 | 13 | (11–16) | 8% |
| | VCR | 0.5 | 11 | 13 | (12–15) | 8% |

[a]Dose in $mg\,kg^{-1}$ i.p. per day for 4 consecutive days. Dosage for both DNR and VCR correspond to LD10 as previously determined by Danø (1971; 1972); [b]Median survival in days after start of treatment. [c]Increased median life span compared to controls.

**Table II** Surface area ratio of CF labelled plasma membrane area to total plasma membrane area, SS(CFm,m)[a]

| *Cell type* | *Median* | *95% CL*[b] | *N*[c] |
|---|---|---|---|
| P388/S/4°C[d] | 0.875 | 0.850–0.900 | 45 |
| P388/S/15[e] | 0.727[k] | 0.690–0.765 | 59 |
| P388/S/45[f] | 0.736[l] | 0.647–0.750 | 54 |
| P388/DNR+/4°C[d] | 0.865 | 0.844–0.875 | 38 |
| P388/DNR+/15[e] | 0.428[k,m] | 0.306–0.455 | 46 |
| P388/DNR+/15/DNR[g] | 0.397 | 0.211–0.482 | 53 |
| P388/DNR+/15/VER[i] | 0.49[m] | 0.346–0.563 | 50 |
| P388/DNR+/45[f] | 0.286[l,n] | 0.235–0.318 | 49 |
| P388/DNR+/45/DNR[h] | 0.326 | 0.194–0.417 | 49 |
| P388/DNR+/45/VER[j] | 0.44[n] | 0.353–0.474 | 45 |

[a]Surface area ratio of CF labelled plasma membrane to total plasma membrane demonstrating a significantly greater decrease at both 15 and 45 min at 37°C in P388/DNR+ compared to P388/S; [b]95% confidence limits of the median; [c]Number of whole cell profiles; [d]15 min at 4°C; [e]15 min at 37°C; [f]45 min at 37°C; [g]15 min at 37°C with 5 $\mu$M DNR; [h]45 min at 37°C with 5 $\mu$M DNR; [i]15 min at 37°C with 25 $\mu$M verapamil; [j]45 min at 37°C with 25 $\mu$M verapamil; [k]$P<0.001$; [l]$P<0.001$; [m]$P<0.05$; [n]$P<0.001$ (Mann–Whitney rank sum test).

coded. Experiments with P388/S and P388/DNR+ cells were carried out simultaneously and repeated after 2 weeks. Thus the total material examined by electron microscopy was 8 blocks (pellets) from each experimental period and cell type.

### *Morphometric procedure*

Morphometric evaluation was carried out using a 5:1 coherent double lattice test system as previously described (Sehested *et al.*, 1987) with a distance between fine lines of 0.501 $\mu$m. The following absolute values (per mean cell) were calculated: the median area of the total plasma membrane surface, the median volume of intracellular CF, V(CFi,c) (Table III), median endosomal volume, V(End,c) (Table IV), median area of CF labelled intracellular membrane, S(CFi,c) (Table V), the median endosomal membrane area, S(End,c) (Table VI) and the median surface area ratio of the CF labelled plasma membrane to the total plasma membrane area, SS(CFm,m) (Table II). Mean cell volumes were determined for each experimental period by measuring the diameters of 300 cells at a magnification of ×1,000.

In addition, the median number of endosomes per mean cell, $N(End,c) = NV(End,c) \times V$, was calculated according to the method of Weibel and Gomez (Weibel, 1979) from the equation $NV = (K/\beta) \times \sqrt{(NA^3/pp)}$ (Table VII), where $NV$ is the number of profiles per unit volume, $NA$ the number of profiles per unit area and $PP$ the number of profile test points divided by the total number of test points. $K$ was determined by the coefficient of variation in each test group. The value of $\beta$ was chosen as that of a sphere (1.38), which is an approximation as endosomes are not truly spherical. However this coefficient varies little for short-axed ellipsoids. Finally, the size distributions of endosome profile diameters were calculated from direct measurements of all endosomal profiles (Figure 4). Endosomes are defined in this context as any clear vesicular intracellular profile, regardless of size, which contains CF and is without the typical dense lysosomal matrix.

For statistical analysis the Mann–Whitney rank sum test (two-tailed for unpaired observations) was used. A $P$ value of $<0.05$ was considered a statistically significant difference.

### Results

The ultrastructural appearance of P388 cells reflects their lymphoid histogenesis in that the cytoplasm has few organelles and instead largely contains free ribosomes (Figures 1–3). Unlabelled P388/DNR+ cells appeared to

**Figure 1** P388/S cell incubated with CF for 45 min at 37°C. Although the surface labelling is distinct, no endocytosed CF is seen in this cell profile. The cytoplasm contains mitochondria, a few cisterns of endoplasmic reticulum, and numerous free ribosomes. ×28,000. Bar = 0.5 $\mu$m.

**Figure 2** P388/DNR+ cell incubated with CF for 45 min at 37°C. Internalized CF is present in some large peripheral endosomes (En). ×28,000. Bar = 0.5 $\mu$m.

**Figure 3** P388/DNR+ cell incubated with CF for 45 min at 37°C. In this micrograph CF is seen in structures localized more to the centre of the cell: a typical small endosome (En), structures appearing as multivesicular bodies (Mvb), and dense bodies (Db) – presumably representing lysosomes. A small unlabelled Golgi complex (Go) is also shown. ×37,000. Bar = 0.5 $\mu$m have more endocytotic structures (vacuoles and multivesicular bodies) than P388/S cells. As also observed in EHR cells (Sehested *et al.*, 1987), P388 cells showed a decrease in cell volume after transfer from 4°C to 37°C with a corresponding rise in the surface to volume ratio of plasma membrane to cell so that the cell surface area was constant (data not shown).

All the morphometric variables used to examine both plasma membrane traffic as well as the endosomal compartment demonstrated a highly significant ($P<0.001$) difference between P388/S and P388/DNR+ cells (as exemplified in Figures 1–3). In fact, the endosomal compartment in P388/S cells was so small as to be below the 'detection level' of the morphometric assay used as regards volume and numerical density determinations, as shown by the median zero values in Tables III, IV and VII. The medians of the variable distributions are therefore supplemented with the distribution range in these tables to show that the endosomal compartment, though small, was present in P388/S cells.

The three variables designating endocytosis, *viz* surface area ratio of CF labelled plasma membrane to total plasma membrane area (Table II), volume of intracellular CF per cell (Table III) and surface area of intracellular CF labelled membrane (Table V) all show a significantly greater rate of endocytosis in P388/DNR+ compared to P388/S. Furthermore, in all three variables, P388/S cells fail to demonstrate further endocytosis after 15 min while P388/DNR+ cells continue to do so, though at a lower rate than during the first 15 min.

On the other hand, the three variables designating the endosomal compartment of the cells, *viz* endosomal volume (Table IV), membrane area of endosomes (Table VI) and endosomal number (Table VII) show no significant difference between 15 and 45 min in either cell line. However, P388/DNR+ cells have a significantly greater endosomal compartment then P388/S cells as evidenced by all three variables. This increase in endosomal compartment in P388/DNR+ cells is not only due to a larger number of endosomes, but also to an increase in the individual endosomal volume as reflected by the distributions of the endosome profile diameters shown in Figure 4.

As shown in Table II, incubation with DNR was without effect on endocytosis in P388/DNR+, while verapamil had an inhibitory influence which was significant at both 15 min ($P<0.05$) as well as at 45 min ($P<0.001$).

**Table III** Volume of intracellular CF per cell, V(CFi,c) ($\mu m^3$)[a]

| *Cell type* | *Median* | *95% CL*[b] | *Range* |
|---|---|---|---|
| P388/S/4°C | 0 | 0–0 | 0– 0 |
| P388/S/15 | 0[c] | 0–0 | 0– 4.10 |
| P388/S/45 | 0[d] | 0–0 | 0–11.12 |
| P388/DNR+/4°C | 0 | 0–0 | 0– 2.25 |
| P388/DNR+/15 | 1.45[c,e] | 0–3.36 | 0–20.16 |
| P388/DNR+/45 | 4.56[d,e] | 3.6–6.8 | 0–27.2 |

[a]Volume of intracellular CF per mean cell in P388/S and P388/DNR+ demonstrating a significantly greater increase in P388/DNR+ compared to P388/S at both 15 and 45 min at 37°C; [b]95%confidence limits of the median; [c]$P<0.001$; [d]$P<0.001$; [e]$P<0.005$ (Mann-Whitney rank sum test). For explanation of cell types see **Table II**.

**Table IV** Endosomal volume per cell, V(End,c) ($\mu m^3$)[a]

| *Cell type* | *Median* | *95% CL*[b] | *Range* |
|---|---|---|---|
| P388/S/4°C | 0 | 0–0 | 0– 1.88 |
| P388/S/15 | 0[c] | 0–0 | 0–15.01 |
| P388/S/45 | 0[d] | 0–0 | 0–11.12 |
| P388/DNR+/4°C | 0 | 0–0 | 0– 2.25 |
| P388/DNR+/15 | 4,71[c,e] | 1.95–7.66 | 0–75.26 |
| P388/DNR+/45 | 7.75[d,e] | 4.56–9.59 | 0–36.2 |

[a]Endosomal volume per mean cell showing a significant difference between P388/S and P388/DNR+ at both 15 and 45 min at 37°C. Note lack of significant difference between P388/DNR+ at 15 and 45 min at 37°C. [b]95% confidence limits of the median; [c]$P<0.001$; [d]$P<0.001$; [e]NS. For explanation of cell types see **Table II**.

**Table V** Surface area of intracellular labelled membrane, S(CFi,c) ($\mu m^2$)[a]

| *Cell type* | *Median* | *95% CL*[b] |
|---|---|---|
| P388/S/4°C | 0 | 0–0 |
| P388/S/15 | 8.4[c,e] | 0–10.7 |
| P388/S/45 | 11.7[d,e] | 0–17.8 |
| P388/DNR+/4° | 0 | 0–0 |
| P388/DNR+/15 | 43.1[c,f] | 24.2–53.1 |
| P388/DNR+/45 | 72.8[d,f] | 52.4–98.6 |

[a]Surface area of intracellular CF labelled membrane per mean cell demonstrating a significantly greater amount in P388/DNR+ cells at both 15 and 45 min at 37°C. Note lack of significant rise in P388/S between 15 and 45 min at 37°C while this is evident in P388/DNR+. [b]95% confidence limits of the median; [c]$P<0.001$; [d]$P<0.001$; [e]NS; [f]$P<0.01$ (Mann-Whitney rank sum test). For explanation of cell types see **Table II**.

**Table VI** Endosomal membrane area per cell, S(End,c) ($\mu m^2$)[a]

| *Cell type* | *Median* | *95% CL*[b] |
|---|---|---|
| P388/S/4°C | 0 | 0–0 |
| P388/S/15 | 14.1[c,e] | 9.6–25.3 |
| P388/S/45 | 17.5[d,e] | 6.1–23.9 |
| P388/DNR+/4°C | 0 | 0–0 |
| P388/DNR+/15° | 73.6[c,f] | 44.4–105.5 |
| P388/DNR+/45 | 95.5[d,f] | 69.4–125.1 |

[a]Endosomal membrane area per mean cell showing a significantly larger area in P388/DNR+ compared to P388/S. Note lack of significant difference between 15 and 45 min at 37°C in both cell lines; [b]95% confidence limits of the median; [c]$P<0.001$; [d]$P<0.001$; [e]NS; [f]NS (Mann–Whitney rank sum test). For explanation of cell types see **Table II**.

**Table VII** Number of endosomes per cell, N(End,c)[a]

| *Cell type* | *Median* | *95% CL*[b] | *Range* |
|---|---|---|---|
| P388/S/4°C | 0 | 0–0 | 0–12 |
| P388/S/15 | 0[c,e] | 0–0 | 0–772 |
| P388/S/45 | 0[d,e] | 0–0 | 0–537 |
| P388/DNR+/4°C | 0 | 0–0 | 0–42 |
| P388/DNR+/15 | 139[c,f] | 81–237 | 0–1953 |
| P388/DNR+/45 | 205[d,f] | 141–269 | 0–2054 |

[a]Number of endosomes per mean cell demonstrating a significant difference between P388/S and P388/DNR+. Note lack of significant difference between 15 and 45 min at 37°C in both cell lines; [b]95% confidence limits of the median. [c]$P<0.001$; [d]$P<0.001$; [e]NS; [f]NS (Mann-Whitney rank sum test). For explanation of cell types see **Table II**.

### Discussion

The present study has focused on 3 aspects of the pleiotropically resistant cell *viz* (1) its capacity for recycling plasma membrane; (2) its endosomal compartment as defined by endosomal number, volume and membrane area; and (3) the influence on plasma membrane traffic by both the drug (DNR) to which the cell was made resistant, as well

**Figure 4** Size distributions of endosome profiles in P388/S and P388/DNR+ at 15 and 45 min at 37°C. Note marked difference between the 2 cell lines but similar distributions at 15 and 45 min in both cell lines. Abscissa = endosomal profile diameters in nm.

as by verapamil, a well known anthracycline resistance modulator in P388 cells (Kessel & Wilberding, 1985; Tsuruo *et al.*, 1982).

P388/DNR+ cells endocytosed significantly more CF than P388/S cells, which is in agreement with the results of Basrur *et al.* (1985) who studied the uptake of fluoresceinated lectins in P388/S and P388 cells resistant to DOX. In contrast to P388/DNR+ cells, P388/S cells showed no increase in endocytosis of CF between 15 and 45 min, indicating that the amount of plasma membrane available for recycling is significantly less in sensitive cells. Thus, after 45 min 14% of the CF coated plasma membrane had been endocytosed in P388/S cells compared to 58% in P388/DNR+ cells (Table II). The low endocytotic rate in P388/S cells agrees well with the findings of Goldmacher *et al.* (1986) who described a low capacity of pinocytosis in 4 lymphoblastic cell lines.

As demonstrated in Tables IV, VI and VII and in Figure 4, the endosomal compartment of P388/DNR+ cells is significantly increased compared to P388/S cells. This is in agreement with recent reports stating that CEM human leukaemia cells resistant to VBL, by light microscopy, have a larger number of lipid negative cytoplasmic vacuoles compared to sensitive cells (Zamora & Beck, 1986), and that DOX resistant human intestinal I-407 cells, by electron microscopy, contain a larger number of transport vesicles near the plasma membrane than do sensitive I-407 cells (Geri *et al.*, 1986). Of particular interest are the results showing a constant endosomal content in P388/DNR+ cells between 15 and 45 min despite the above mentioned evidence of increased endocytosis during this period. These findings, together with the measurements showing a constant cell surface area during the experiments (data not shown) indicate that (1) the plasma membrane is recycling as would be expected from other studies (Mellman, 1984); (2) that CF, in itself, hardly significantly stimulates endocytosis (as evidenced by the very modest rate of endocytosis in P388/S); and (3) that the increased capacity to recycle plasma membrane in P388/DNR+ is not solely due to an increase in speed of vesicular traffic (in which case the endosomal content would be equal in the two cell lines).

One of the features of the pleiotropic resistance phenotype is the ability of resistant cells to extrude the drug in question in an energy dependent manner (Skovsgaard & Nissen, 1982). However, the significance and nature of this feature have yet to be determined. We have previously reported that membrane traffic between the plasma membrane and the endosomal system was significantly increased in 4 pleiotropic resistant EHR cell lines compared to the sensitive EHR line (Sehested *et al.*, 1987), and hypothesized that this phenomenon could offer an explanation of the increased active drug extrusion in resistant cells via drug trapping in the endosomal system and exocytosis therefrom to the extracellular medium. A possible method of drug trapping in the acid endosomal compartment could be by protonation of the respective alkaline anthracycline and vinca alkaloid drugs. In support of a pH-dependent drug trapping mechanism are reports on the circumvention of pleiotropic resistance by such dissipators of the endosomal proton gradient as chloroquine and methylamine (Klohs & Steinkampf, 1986; Shiraishi *et al.*, 1986; Zamora & Beck, 1986), as well as the inhibition of drug extrusion in resistant cells by chloroquine (Shiraishi *et al.*, 1986). Furthermore, DOX is rapidly trapped in unilamellar vesicles in response to a transmembrane pH gradient with an acidic interior, with a trapping efficiency up to 98% and an interior drug concentration as high as 100 mM (Mayer *et al.*, 1986). However, Cornwell *et al.* (1986) found that plasma membrane and endocytotic vesicles prepared from pleiotropically resistant KB cells bound up to

8 fold more VBL than did vesicles from parental or revertant KB cells in a trypsin dependent, $NH_4Cl$ independent fashion, signifying the additional possibility of drug trapping by a protein receptor. A likely candidate for such a protein would be the P-glycoprotein described by Juliano & Ling (1976), which is commonly overexpressed in highly resistant *in vitro* cell lines, and has been detected both in the above mentioned resistant KB line (Shen *et al.*, 1986) as well as in a DOX resistant P388 *in vitro* cell line (Kessel & Corbett, 1985). Whether P-glycoprotein is also overexpressed in our *in vivo* resistant cell lines, which are of relatively low resistance compared to *in vitro* lines, is currently under investigation.

Studies by Kessel & Wilberding (1985) and Tsuruo *et al.* (1982) have demonstrated that calcium channel blockers such as verapamil, as well as calmodulin inhibitors, are able to reverse pleiotropic resistance, and inhibit active DNR efflux in DOX resistant P388 cells by an as yet undetermined mechanism. We have shown that verapamil, in a dose that nearly completely inhibits active DNR efflux in both DOX resistant P388 cells (Kessel & Wilberding, 1985), as well as in DNR resistant EHR cells (Friche *et al.*), also significantly inhibits plasma membrane traffic in P388/DNR+. Calmodulin inhibitors have also been described to decrease vesicular traffic (Kuratomi *et al.*, 1986; Salisbury *et al.*, 1980). Further, Tsuruo & Iida (1986) found that cytochalasin, another drug which is presumed to disrupt vesicular traffic, inhibited the outward transport of DNR from DOX resistant P388 cells.

Thus, the present study supports a role for increased plasma membrane flow and endosomal content in pleiotropic resistance.

We thank Dr Bo Hanau for helpful discussion and Ms Bodil Collatz, Ms Marianne Dam, Ms Marianne Knudsen, Mr Keld Ottosen and Ms Kirsten Pedersen for expert technical assistance.

Supported in part by the Danish Cancer Society and the J. Lauritzen Foundation.

### References

BASRUR, V.S., CHITNIS, M.P. & MENON, R.S. (1985). Cell surface alterations in murine leukaemia P388 adriamycin-resistant cells: Studies on lectin-induced agglutination and rearrangement of lectin receptors. *Oncology*, **42**, 328.

CORNWELL, M.N., GOTTESMAN, M.M. & PASTAN, I.H. (1986). Increased vinblastine binding to membrane vesicles from multidrug-resistant KB cells. *J. Biol. Chem.*, **261**, 7921.

DANØ, K. (1971). Development of resistance to daunomycin (NSC-82151) in Ehrlich ascites tumor. *Cancer Chemother. Rep.*, **55**, 133.

DANØ, K. (1972). Cross resistance between vinca alkaloids and anthracyclines in Ehrlich ascites tumor *in vivo*. *Cancer Chemother. Rep.*, **56**, 701.

DAWE, C.J. & POTTER, M. (1957). Morphologic and biologic progression of a lymphoid neoplasm of the mouse *in vivo* and *in vitro*. *Amer. J. Pathol.*, **33**, 603 (*Abstract*).

EHRLICH, P. & APOLANT, H. (1905). Beobachtungen über maligne Mäusetumoren. *Berliner Klin. Wschr.*, **42**, 871.

FRICHE, E., SKOVSGAARD, T. & NISSEN, N.I. (1987). Effect of verapamil on daunorubicin accumulation in Ehrlich ascites tumor cells. *Cancer Chemother. Pharmacol.*, **19**, 35.

GERI, O., GRANDI, M., BELLINI, O. & 3 others (1986). Characterization of a human intestinal cell line (I-407). *Fifth NCI-EORTC symposium on new drugs in cancer therapy*, 3.09 (*Abstract*).

GOLDMACHER, V.S., TINNEL, N.L. & NELSON, B.C. (1986). Evidence that pinocytosis in lymphoid cells has a low capacity. *J. Cell Biol.*, **102**, 1312.

INABA, M., KOBAYASHI, H., SAKURAI, Y. & JOHNSON, R.K. (1979). Active efflux of daunorubicin and adriamycin in sensitive and resistant sublines of P388 leukemia. *Cancer Res.*, **39**, 2200.

JULIANO, R.L. & LING, V. (1976). A surface glycoprotein modulating drug permeability in Chinese hamster ovary cell mutants. *Biochim. Biophys. Acta*, **455**, 152.

KESSEL, D. & CORBETT, T. (1985). Correlations between anthracycline resistance, drug accumulation and membrane glycoprotein patterns in solid tumours of mice. *Cancer Lett.*, **28**, 187.

KESSEL, D. & WILBERDING, C. (1985). Anthracycline resistance in P388 murine leukemia and its circumvention by calcium antagonists. *Cancer Res.*, **45**, 1687.

KLOHS, W.D. & STEINKAMPF, R.W. (1986). Intrinsic resistance of colon tumors to anthrapyrazoles and anthracyclines may be linked with a detoxification mechanism of intestinal cells. *Proc. Amer. Assoc. Cancer Res.*, **27**, 395 (*Abstract*).

KURATOMI, Y., AKIYAMA, S.-I., ONO, M. & 4 others (1986). Thioridazine enhances lysosomal accumulation of epidermal growth factor with pseudomonas exotoxin. *Exp. Cell Res.*, **162**, 436.

MAYER, L.D., BALLY, M.B. & CULLIS, P.R. (1986). Uptake of adriamycin into large unilamellar vesicles in response to a pH gradient. *Biochim. Biophys. Acta*, **857**, 123.

MELLMAN, I. (1984). Membrane recycling during endocytosis. In *Lysosomes in biology and pathology*, Dingle, J.T. *et al.* (eds) p.210. Elsevier Science Publishers B.V.: Amsterdam.

SALISBURY, J.L., CONDEELIS, J.S. & SATIR, P. (1980). Role of coated vesicles, microfilaments, and calmodulin in receptor-mediated endocytosis by cultured B lymphoblastoid cells. *J. Cell Biol.*, **87**, 132.

SEHESTED, M., SKOVSGAARD, T., VAN DEURS, B. & WINTHER-NIELSEN, H. (1987). Increase in non-specific adsorptive endocytosis in anthracycline and vinca alkaloid resistant Ehrlich ascites tumor cell lines. *J. Natl Cancer Inst.*, **78**, 171.

SHEN, D.-W., CARDARELLI, C., HWANG, J. & 5 others. (1986). Multiple drug-resistant human KB carcinoma cells independently selected for high-level resistance to colchicine, adriamycin, or vinblastine show changes in expression of specific proteins. *J. Biol. Chem.*, **261**, 7762.

SHIRAISHI, N., AKIYAMA, S.-I., KOBAYASHI, M. & KUWANO, M. (1986). Lysomotropic agents reverse multiple drug resistance in human cancer cells. *Cancer Lett.*, **30**, 251.

SKOVSGAARD, T. & NISSEN, N.I. (1982). Membrane transport of anthracyclines. *Pharmac. Ther.*, **18**, 293.

TSURUO, T., IIDA, H., TSUKAGOSHI, S. & SAKURAI, Y. (1982). Increased accumulation of vincristine and adriamycin in drug-resistant P388 tumor cells following incubation with calcium antagonists and calmodulin inhibitors. *Cancer Res.*, **42**, 4370.

TSURUO, T. & IIDA, H. (1986). Effects of cytochalasins and colchicine on the accumulation and retention of daunomycin and vincristine in drug resistant tumor cells. *Biochem. Pharmac.*, **35**, 1087.

WEIBEL, E.R. (1979). Stereological methods. Vol. 1. *Practical methods for biological morphometry*. Academic Press: London.

ZAMORA, J.M. & BECK, W.T. (1986). Chloroquine enhancement of anti-cancer drug cytotoxicity in multiple drug resistant human leukemic cells. *Biochem. Pharmac.*, **35**, 4303.